# Developing Gaussian process regression, Lasso regression, and Nu-support vector regression models for predicting solubility of exemestane in supercritical CO_2_

**DOI:** 10.1038/s41598-025-31291-9

**Published:** 2025-12-08

**Authors:** Jawza A. Almutairi, Thamir Malik

**Affiliations:** 1 Department of Pharmaceutical Sciences, College of Pharmacy, Princess Nourah bint Abdulrahman University, P.O. Box 84428, 11671 Riyadh, Saudi Arabia; 2 Karbala Refinery, Midland Refineries Company, Ministry of Oil, Karbala, 56001 Iraq; 3 Oil and Gas Engineering Department, University of Warith Al-Anbiyaa, Karbala, 56001 Iraq

**Keywords:** Drug solubility, Supercritical CO_2_, Gaussian process regression, Lasso regression, Nu-Support vector regression, Machine learning, Pharmaceutical processing, Exemestane, Cancer, Chemistry, Computational biology and bioinformatics, Drug discovery, Mathematics and computing

## Abstract

Precise estimation of pharmaceutical solubility in supercritical carbon dioxide (scCO_2_) is essential for optimizing pharmaceutical applications, including particle size reduction, the development of solid dispersions, and controlled-release formulations. In this research, we present a comparative analysis of three machine learning regression models—Lasso Regression, Gaussian Process Regression (GPR), and Nu-Support Vector Regression (Nu-SVR)—for predicting the solubility of exemestane (EXE), a poorly water-soluble anticancer drug, in scCO_2_ under varying temperature and pressure conditions. The dataset used in this work consists of 45 experimental measurements encompassing temperature (T in K), pressure (P in MPa), and solubility (in g/L) of EXE. The dataset was divided into training and testing data subsets to facilitate reliable model validation. Model performance was thoroughly evaluated using metrics such as the R², RMSE, MAE, and AARD%. Additionally, decision surfaces and observed-versus-predicted plots were generated to visually assess model accuracy. Among the applied models, Gaussian Process Regression demonstrated superior predictive capability with an R² score of 0.996, Maximum error of 3.27, significantly outperforming both Lasso and Nu-SVR models. These results indicate that GPR effectively captures the nonlinear relationship between process variables and drug solubility, offering high generalization and precision. Feature importance analysis confirmed that pressure has the most significant influence on solubility behavior, while temperature also contributes positively to solubility trends. Residual analysis further validated the consistency and reliability of the GPR-based model. This work contributes to the growing application of machine learning techniques in pharmaceutical process modeling, particularly in supercritical fluid-based drug delivery systems. The proposed GPR model provides a reliable and efficient tool for predicting solubility, supporting the design and optimization of scCO_2_-assisted drug formulation methods.

## Introduction

Multi-particulate dosage forms have been considered as a breakthrough technology in the pharmaceutical industry thanks to their remarkable potential as a drug delivery system with disparate applications^1,2^. In the current decades, the ultimate purpose of formulation scientists all over the world have been focused on the development of promising state-of-the-art technologies to rise the efficiency and bioavailability of orally-administered drugs with minimum systemic toxicity. Development of such technologies not only provides significant hopes for those patients suffering from different fatal diseases but also can propose excellent chances to the industries to enhance their market share, particularly for competitive therapeutic agents^3–5^.

According to the biopharmaceutics classification system (BCS), principal parameters for describing the absorption behavior of the orally-administered therapeutic agents are solubility and permeability^6^. Therefore, finding cost-effective and green paradigm to optimize and increase the solubility of poorly-soluble lipophilic drugs in water is a remarkable aim in the pharmaceutical industry^7^. Application of CO_2_ supercritical fluid (CO_2_-SCF) for increasing the solubility of poorly-soluble therapeutic agents in water has recently attracted the attentions of scientists^8–10^. CO_2_ in the normal condition is considered as one of the most important greenhouse gases. Therefore, some topics such as global warming and air pollution may be raised^11,12^. However, the use of CO_2_ can be an efficient method to enhance the solubility of orally-administered drugs with low solubility in water due to its considerable advantages like great potential to manufacture particles with appropriate aerodynamic diameters, very low need of heating to produce the fundamental particles, low toxicity, safety and available critical conditions^13,14^.

Exemestane (Aromasin, with chemical formula C20H24O2) is a commonly-used steroidal aromatase inhibitor, which has been indicated by the U.S food and drug administration (FDA) since October 1999 for the adjuvant treatment of postmenopausal female patients suffering from hormonally-responsive breast cancer in women. Exemestane significantly declines the production of estrogen by the body and therefore, stop the growth of those cancerous cells that are sensitive to estrogen^15–17^.

Machine learning (ML) methodologies are gradually replacing traditional computing methods in a variety of scientific disciplines^18,19^. Neural Networks, Deep Learning, Linear Models and ensemble methods are examples of these approaches that are used to solve a variety of problems such as energy, fluid properties, materials, separation, etc^20,21^. Machine learning models may now analyze any problem by providing certain input qualities and single or multiple target outputs. These models capture the relationships between inputs and outputs through different mechanisms^22^.

It is common practice to employ SVR, an algorithm grounded in statistical learning theory, to enhance generalization capacity. Based on their estimation function, support vector-based models have numerous versions. There are many support vector regression varieties. We employed Nu-SVR model in this study^23,24^. Moreover, the Gaussian process model (GPR) is an effective non-parametric Bayesian model for exploration and exploitation. The primary benefit of GPR the ability to process a consistent answer for the model’s input properties^25^. Bayesian inference is used to let the data determine the complexity of the models, allowing this approach to portray an extensive variety of correlations between input qualities and output values^26–29^. Linear regression is a popular statistical analysis method. It’s fundamental, but it’s incredibly useful in places like economics, material science, and chemistry. As another linear model, LASSO regression is often used. The Lasso is a linear model for sparse coefficient prediction^30^. In this study, the solubility of Exemestane at various temperatures and pressures in supercritical CO_2_ (scCO_2_) was evaluated using several novel mathematical models developed through artificial intelligence techniques. Comparative analysis indicated that Gaussian Process Regression (GPR) provided the most accurate predictions, achieving the highest R² value (0.996) alongside the lowest error rate (MAE = 0.904).

## Data set for computing

**Fig. 1:**
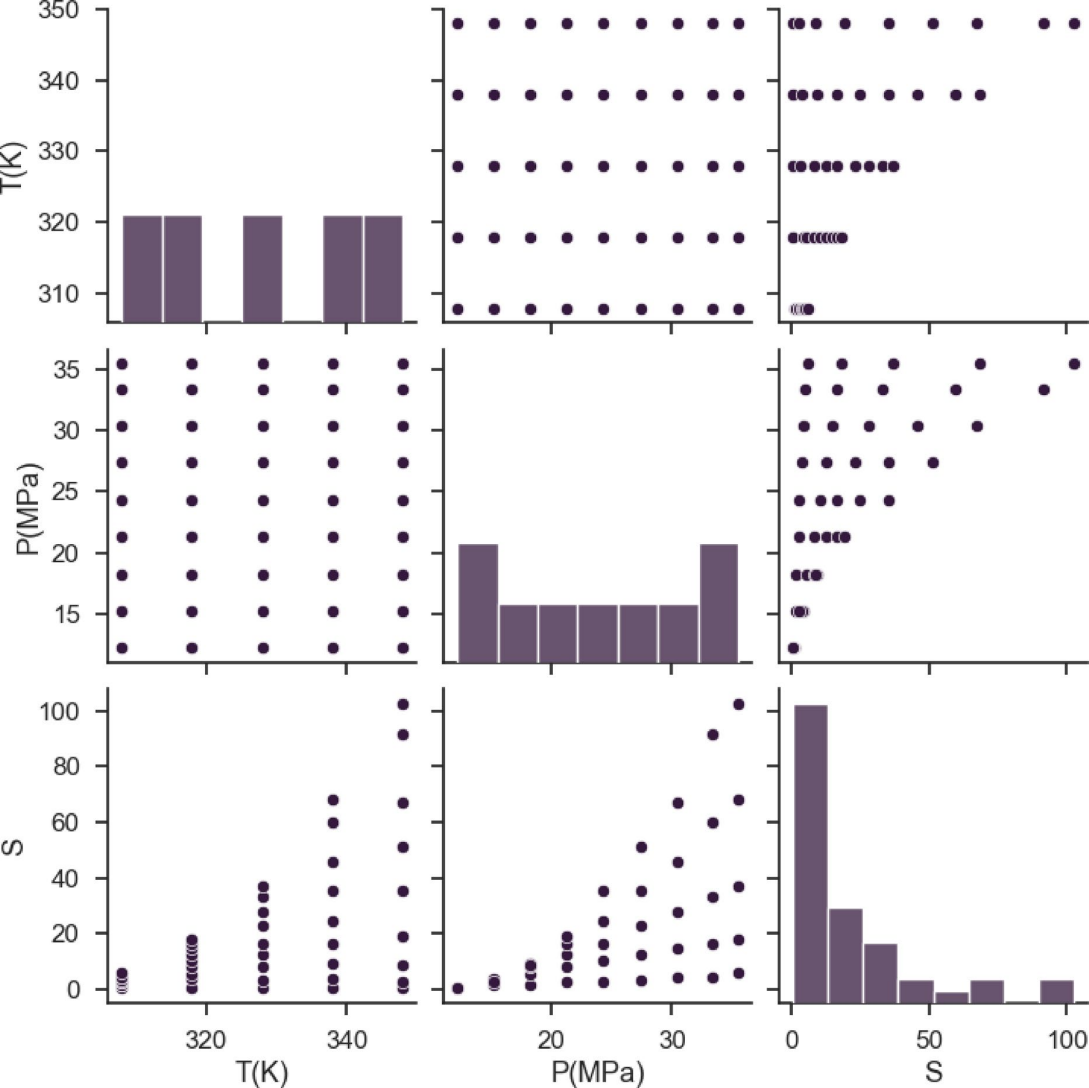
Distributions of parameters.

This study involves a regression task. This task contains 45 data points, that are organized below in the table: Two numerical inputs of T (K) and P (MPa) and a single numerical output are provided (Solubility of EXE drug). Table 1 shows the dataset of our research (taken from^31^ which can be accessed using: https://www.sciencedirect.com/science/article/pii/S0896844609002071. Figure 1 shows the pairwise distribution of variables.

**Table 1:** The whole used dataset^31^.

T (K)	*P* (MPa)	S (×10 g L^−1^)
308	12.2	0.67
	15.2	1.38
	18.2	1.47
	21.3	2.41
	24.3	2.5
	27.4	3.41
	30.4	4.1
	33.4	4.55
	35.5	5.92
318	12.2	0.56
	15.2	4.01
	18.2	5.13
	21.3	8.23
	24.3	10.39
	27.4	12.33
	30.4	14.78
	33.4	16.47
	35.5	17.78
328	12.2	0.53
	15.2	3.25
	18.2	8.23
	21.3	12.28
	24.3	16.46
	27.4	22.85
	30.4	28.02
	33.4	33.18
	35.5	36.94
338	12.2	0.35
	15.2	3.82
	18.2	8.99
	21.3	16.57
	24.3	24.65
	27.4	35.36
	30.4	45.7
	33.4	59.79
	35.5	68.25
348	12.2	0.34
	15.2	2.5
	18.2	8.42
	21.3	19.2
	24.3	35.36
	27.4	51.34
	30.4	67.31
	33.4	91.74
	35.5	102.67

## Methodology

### Cuckoo search algorithm (CS)

The CS algorithm^32,33^ is a meta-heuristic optimizer which operates based on swarm intelligent. Rhododendron homing parasitic characteristics are simulated^34^. Levy flight is used to get the best possible incubation conditions for a host species’ eggs anywhere in the world. CS algorithm follows these three principles^34–36^:

1. There is only ever one egg laid by a cuckoo at a time, and the nesting sites are selected at random.
2. Among a randomly chosen group of nests, only the nest with the superior-quality eggs is selected to generate the next generation.
3. In every generation of cuckoos, the number of available host nests remains constant^34^.

### GPR

Probabilistic regression has the potential to increase robustness to learning mistakes in many cases. Methods for nonlinear regression that rely on a probabilistic regression framework but non-parametric models^37^; examples include GPR (Gaussian Process Regression). The premise of this approach is that the y measurements that constitute the output variable are generated as follows^25^:

$$\:y=f\left(\mathbf{x}\left(k\right)\right)+\epsilon\:$$

$\:{\:\sigma\:}_{n}^{2}\:$is the Gaussian noise variance. Instead of giving parameters to the function *f*, the prior probability is described in respect of the GP, which applies across the entire function space^38^. The mean *m(x)* and the covariance equation *cov(x*, *x*′*)* of the GP carry ideas about the generating mechanism. The covariance and mean equations are computed, and there we can derive the output corresponding to a specific data point *x* based on Gaussian distribution *p(y*_∗_*|X*,* y*,* x*_∗_*)* with^39^:

$$\:\begin{array}{cc}&\:{\stackrel{{}^{}}{y}}_{*}=m\left({\mathbf{x}}_{*}\right)+{\mathbf{k}}_{*}^{\text{T}}{\left(\mathbf{K}+{\sigma\:}_{n}^{2}\mathbf{I}\right)}^{-1}\left(\mathbf{y}-m\left({\mathbf{x}}_{*}\right)\right)\text{,}\\\:&\:{\sigma\:}_{{y}_{*}}^{2}={k}_{*}+{\sigma\:}_{n}^{2}-{\mathbf{k}}_{*}^{\text{T}}{\left(\mathbf{K}+{\sigma\:}_{n}^{2}\mathbf{I}\right)}^{-1}{\mathbf{k}}_{*}\text{,}\end{array}$$

Following the equation presented above, an estimate is calculated on the train vector *X*,* y*. Conversely, the prediction in conventional regression methods is based solely on the parameters.

In this formula, *K* represents a covariance matrix which the elements in this matrix are *K*_*i, j*_ *= cov(x*_*i*_, *x*_*j*_*)*, and k is a vector^40^:

$$\:\left[k*\right]i=cov\left(xi,x*\right)andk*=cov\left(x*,x*\right)$$

The variables of the mean and covariance functions need has been computed through dataset before reliable predictions can be made. Optimizing log *p(y|X)*, the log likelihood amount of the train subset, is typically applied for organizing the hyper-variables^41^:

$$\:\text{l}\text{o}\text{g}p\left(\mathbf{y}|\mathbf{X}\right)=-\frac{1}{2}{\mathbf{y}}^{\text{T}}{\left(\mathbf{K}+{\sigma\:}_{n}^{2}\mathbf{I}\right)}^{-1}\mathbf{y}-\frac{1}{2}\text{l}\text{o}\text{g}\left(\left|\mathbf{K}+{\sigma\:}_{n}^{2}\mathbf{I}\right|\right)-\frac{n}{2}\text{l}\text{o}\text{g}\left(2\pi\:\right)$$

In the recent equation, *n* shows the quantity of instances in the training subset.

### Nu-SVR

The Support Vector Machines (SVM) approach fundamentally tries to map the input data vector into a higher dimensional feature space in order to generate an ideal separation hyperplane. By seeing the hyperplane as a curve tube, SVM was effectively used to regression and time series prediction^42,43^. Consider the following input and output values as basic assumptions^43^:

$$\:\left[\left({x}_{1},{y}_{1}\right),\dots\:,\left({x}_{n},{y}_{n}\right)\right]$$

Finding the nonlinear correlation shown by the following Equation, as *f(x)*. The aim of the Nu-SVR model is to have it be as near to *y* as possible. Moreover, it needs to be as level as possible^43^:

f(x) = w^T^ P(x) + b.

$$\text{f}(\text{x})=\text{w}^{\text{T}} \text{P}(\text{x})+\text{b}$$

To clarify, *b* stands for the bias, *w*^*T*^ shows the weight vector, and *P(x)* is a non-linear mapping equation that transforms the feature space into one with more downgrades^25^. The primary focus of the assignment to satisfy the two fundamental requirements of closeness and flatness. In fact, optimizing is the task’s main goal^44^:

$$\frac{1}{2}{\left| {\left\lceil w \right\rceil } \right|^2} + {\text{C}}\left\{ {Y \cdot \varepsilon + \frac{1}{n}\sum\limits_{i = 1}^n {\left( {\xi + \xi *} \right)} } \right\}.$$

Under the following conditions^44^:

$$\text{y}_\text{i{-}} \left\langle {w}^{T}.P\left(x\right) \right\rangle -b\le\:\varepsilon\:+{\xi\:}_{i}^{*},$$

$$\:\left\langle {w}^{T}.P\left(x\right) \right\rangle +b-{y}_{i}\le\:\varepsilon\:+{\xi\:}_{i},$$

$$\:{\xi\:}_{i}^{*},{\xi\:}_{i}\ge\:0$$

Here, ɛ stands for a disparity of the *f(x)* from its experimental data, and extra slack variables^25^ (*ξ*, and *ξ*_*i*_ are) declared in^44^.

### Lasso

The method of LASSO promotes sparsity in coefficient estimates. By favoring solutions with fewer non-zero coefficients, it effectively reduces the number of features contributing to the model, enhancing its applicability in certain scenarios. Compressed sensing relies heavily on models, and Lasso and Lasso-based models are a major part of it. The exact set of coefficients can be found in some situations^45^. This technique is employed to simplify the model and forestall over-fitting. To adjust the residual sum of squares, we choose *β*_*j*_ in the following equation^45^:

$$\:\begin{array}{c}\sum\:_{i=1}^{n}{\left({\beta\:}_{0}+\sum\:_{k=j}^{K}{\beta\:}_{k}{x}_{k,i}-{y}_{i}\right)}^{2}\end{array}$$

*λ* is used in LASSO regression to optimize the sum of the residual squares^45^:

$$\:\begin{array}{c}\sum\:_{i=1}^{n}{\left({\beta\:}_{0}+\sum\:_{k=1}^{K}{\beta\:}_{k}{x}_{k,i}-{y}_{i}\right)}^{2}+\lambda\:\sum\:_{k=1}^{k}{\beta\:}_{k}\:\:\end{array}$$

### Evaluation metrics

The predictive capability of the developed models was evaluated via four standard statistical metrics: the coefficient of determination (R²), mean absolute error (MAE), mean absolute percentage error (MAPE), and maximum error (Max Error). These indicators provide a quantitative assessment of the accuracy and reliability of the predicted solubility values relative to the experimental measurements.

The R² measures how well the predicted values approximate the actual observations and is given by^46^:

$$\:{R}^{2}=1-\frac{{\sum\:}_{i=1}^{n}{\left({y}_{i}-\widehat{y}*i\right)}^{2}}{\sum\:*i={1}^{n}{\left({y}_{i}-\bar{y}\right)}^{2}}$$

MAE measures the average size of prediction errors, indicating the typical deviation of the predicted values from the observed data:

$$\:\text{MAE}=\frac{1}{n}{\sum\:}_{i=1}^{n}\left|{y}_{i}-\widehat{{y}_{i}}\right|$$

The MAPE expresses the average relative error as a percentage:

$$\:\text{MAPE}=\frac{100}{n}{\sum\:}_{i=1}^{n}\left|\frac{{y}_{i}-\widehat{{y}_{i}}}{{y}_{i}}\right|$$

The Max Error determines the largest deviation between experimental and predicted values:

$$\:\text{Max\:Error}=\text{max}\left|{y}_{i}-\widehat{{y}_{i}}\right|$$

In these expressions, $\:{y}_{i}$ and $\:\widehat{{y}_{i}}$ stands for the experimental and calculated solubility values, respectively; $\:\bar{y}$ represents the mean of the observed values, and *n* corresponds to the total number of data points.

## Results and discussions

The introduced models were optimized using the CS algorithm and their effective hyper-parameters were obtained for optimal implementation. At the end, the approaches have been analyzed and validated, then the results of multiple statistical metrics are displayed in Table 2.

The final optimized hyperparameters for all regression models were determined using the CS optimization algorithm to ensure the best predictive performance. For the GPR model, the optimized settings included a squared exponential kernel, kernel scale of 1.42, signal variance of 0.85, and noise level of 0.003. The Nu-SVR model achieved its optimal configuration with a radial basis function (RBF) kernel, penalty parameter C equal to 110, insensitivity parameter epsilon of 0.08, and nu value of 0.45. For the Lasso Regression model, the optimal regularization coefficient lambda was 0.007 with a tolerance value of 1 × 10⁻⁴. These optimized hyperparameter values were obtained based on minimizing the root mean square error and maximizing the coefficient of determination during the cross-validation process, confirming the reliability and generalization capability of the trained models.

**Table 2:** The outputs of final optimized approaches.

Models	*R*^2^ score	MAE	MAPE	Max error
GPR	0.996	0.904	0.064	3.27
Nu-SVR	0.793	5.310	0.873	15.74
LASSO	0.983	1.921	0.115	5.57

**Fig. 2:**
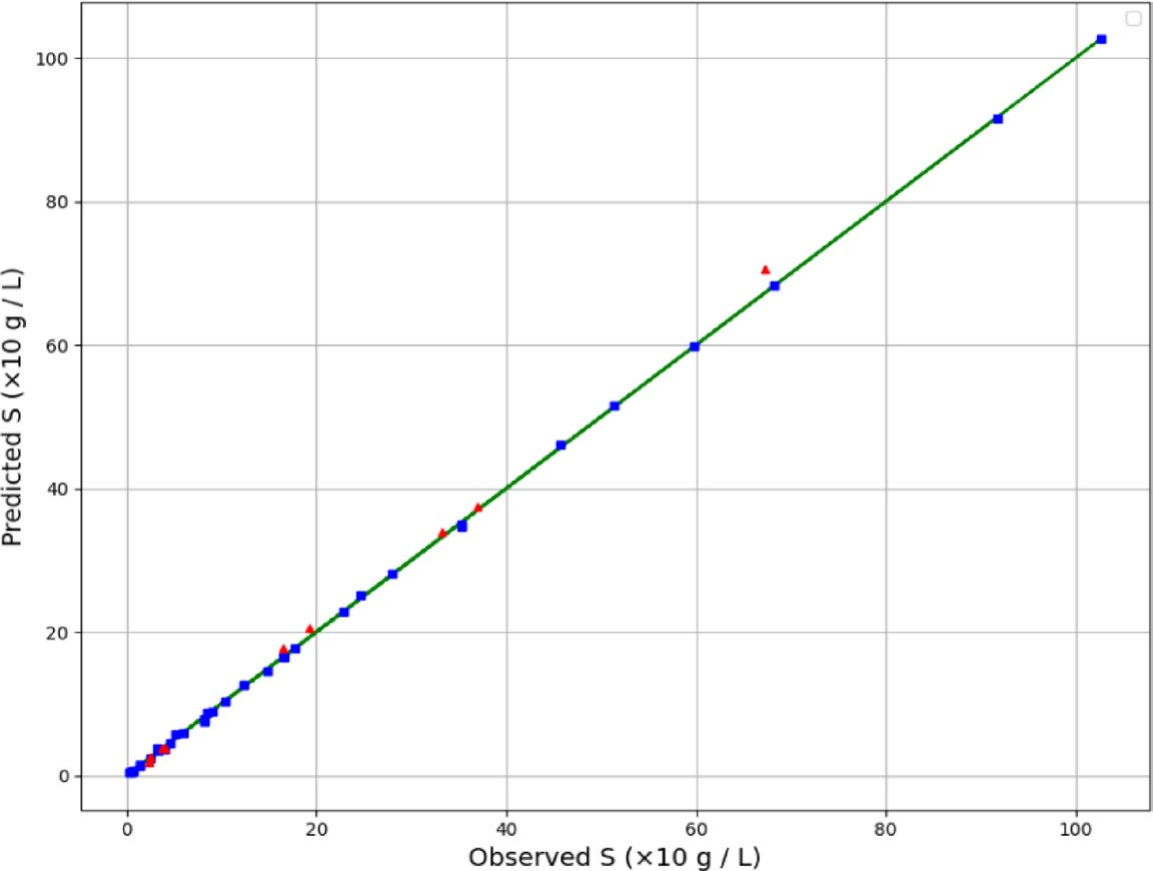
Comparing Observed and estimated output (GPR method).

**Fig. 3:**
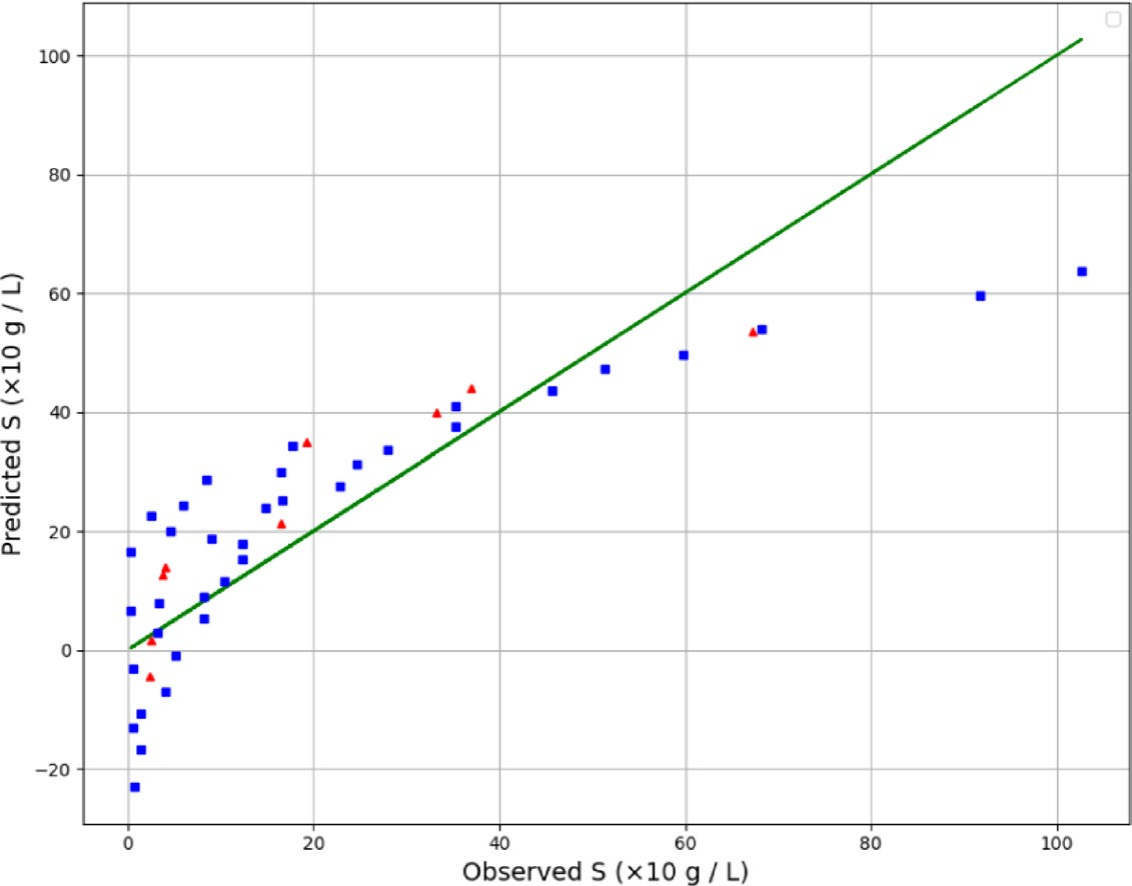
Comparing observed and estimated output (Lasso method).

**Fig. 4:**
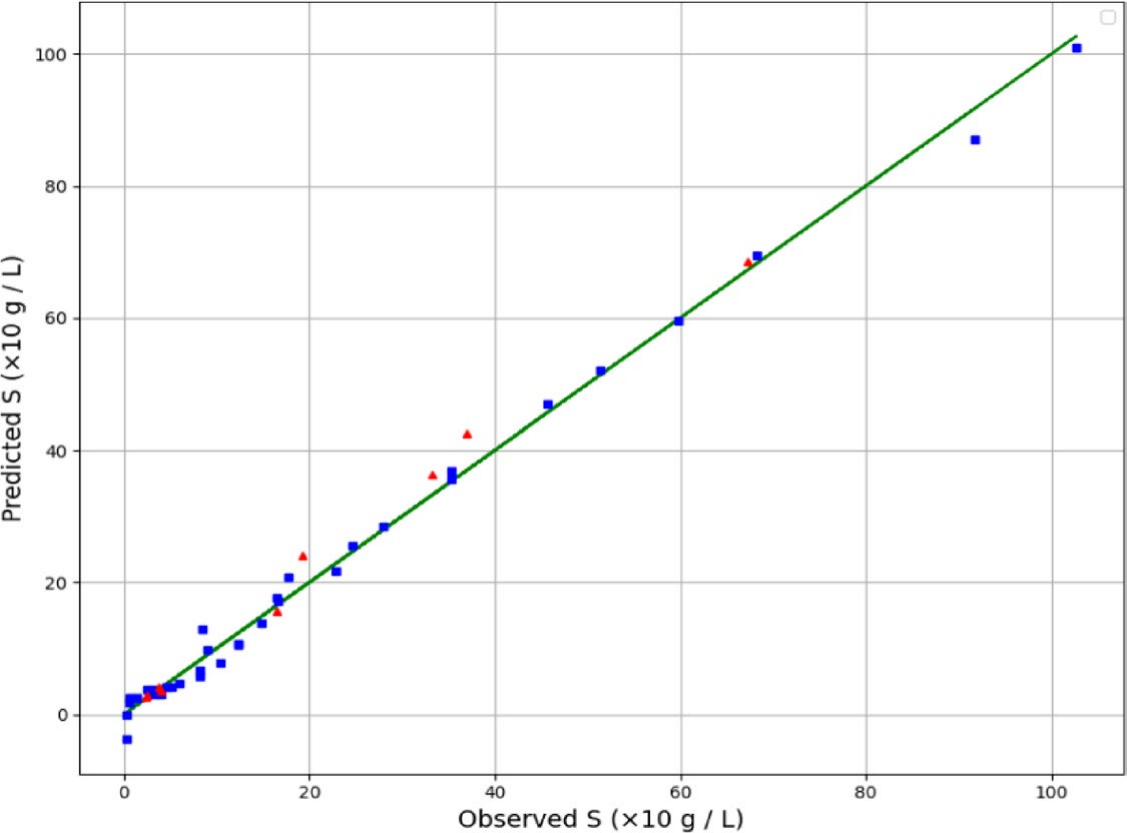
Comparing observed and estimated output (Nu-SVR method).

**Fig. 5:**
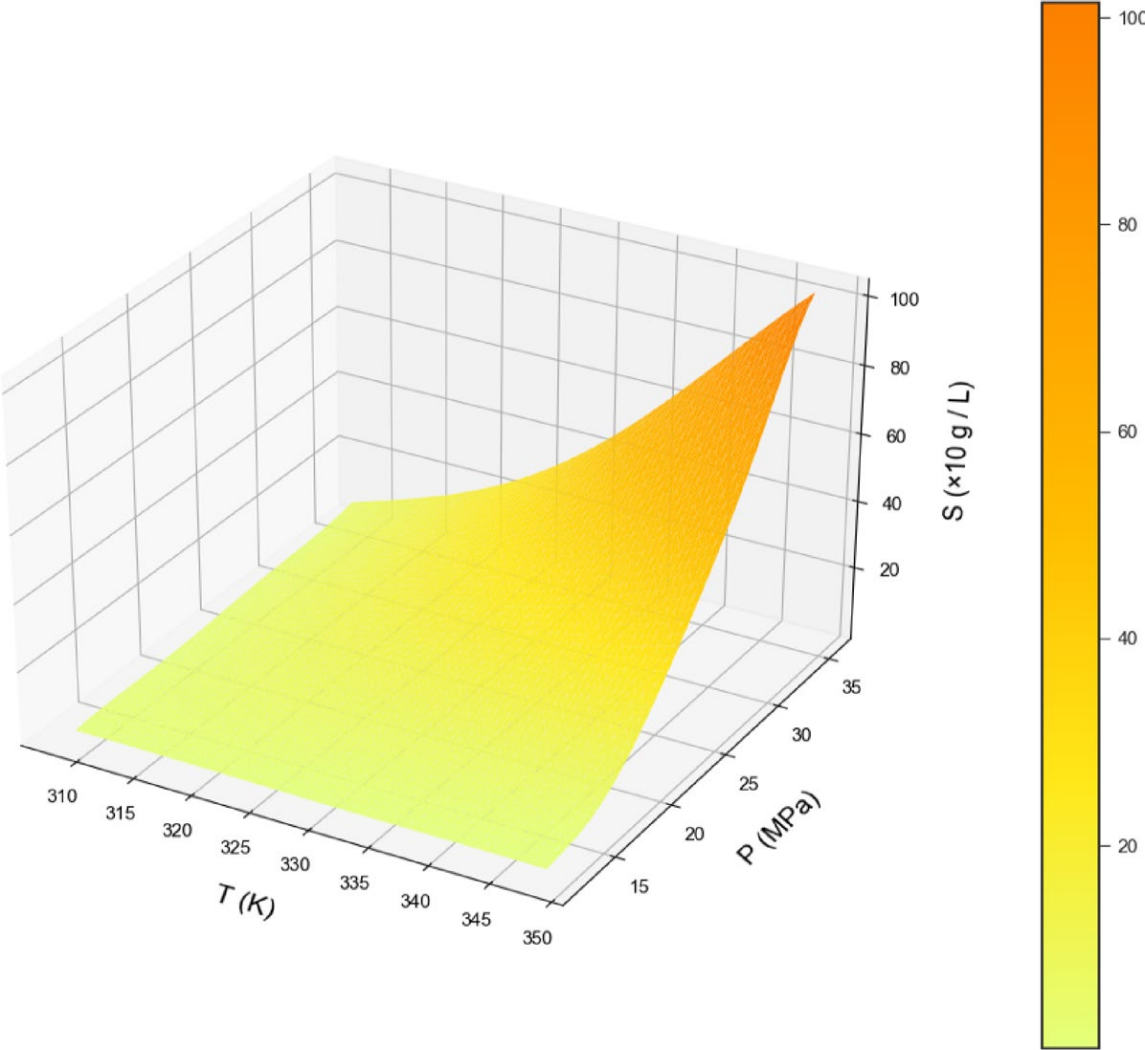
The 3D final decision surface (GPR MODEL).

**Fig. 6:**
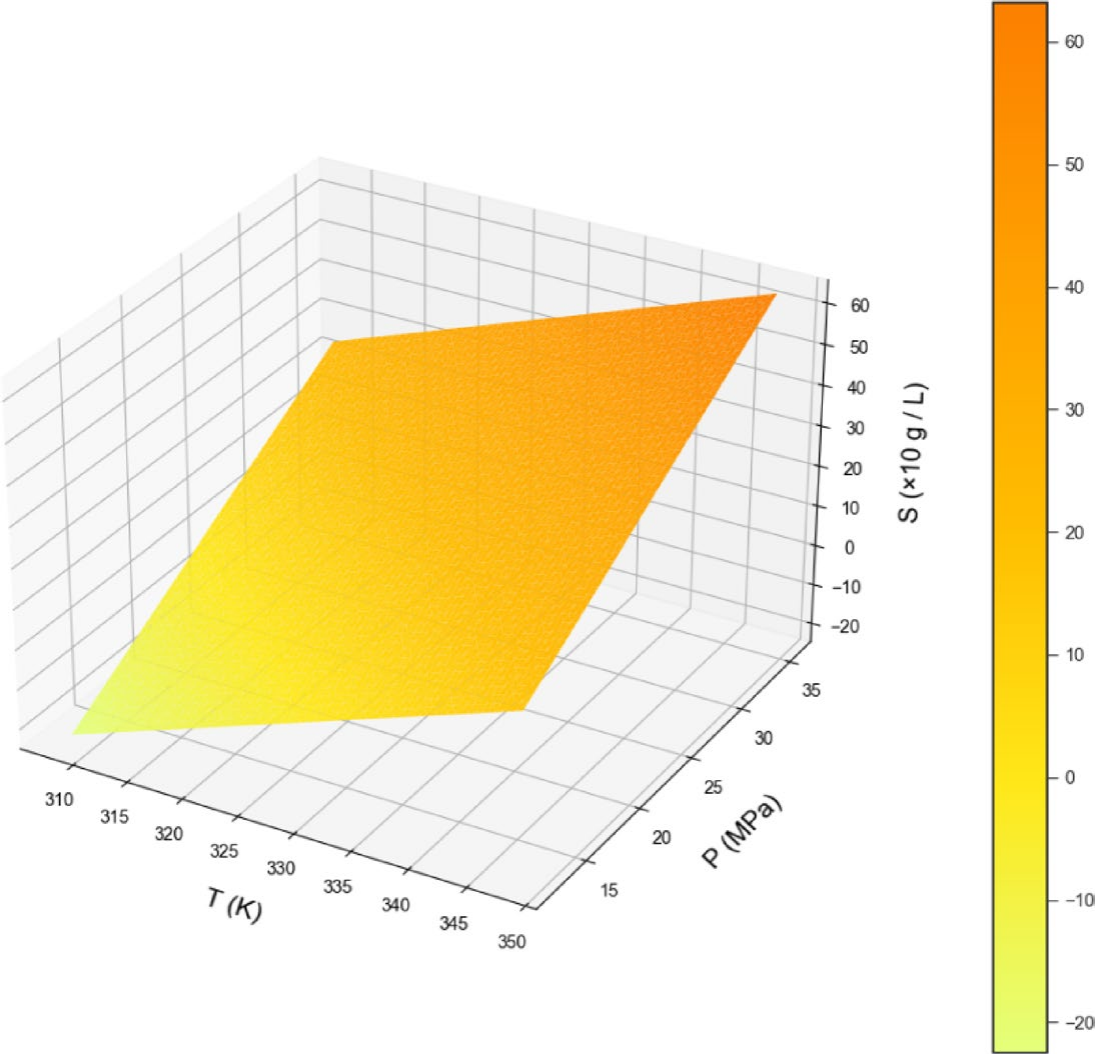
The 3D final decision surface (LASSO MODEL).

**Fig. 7:**
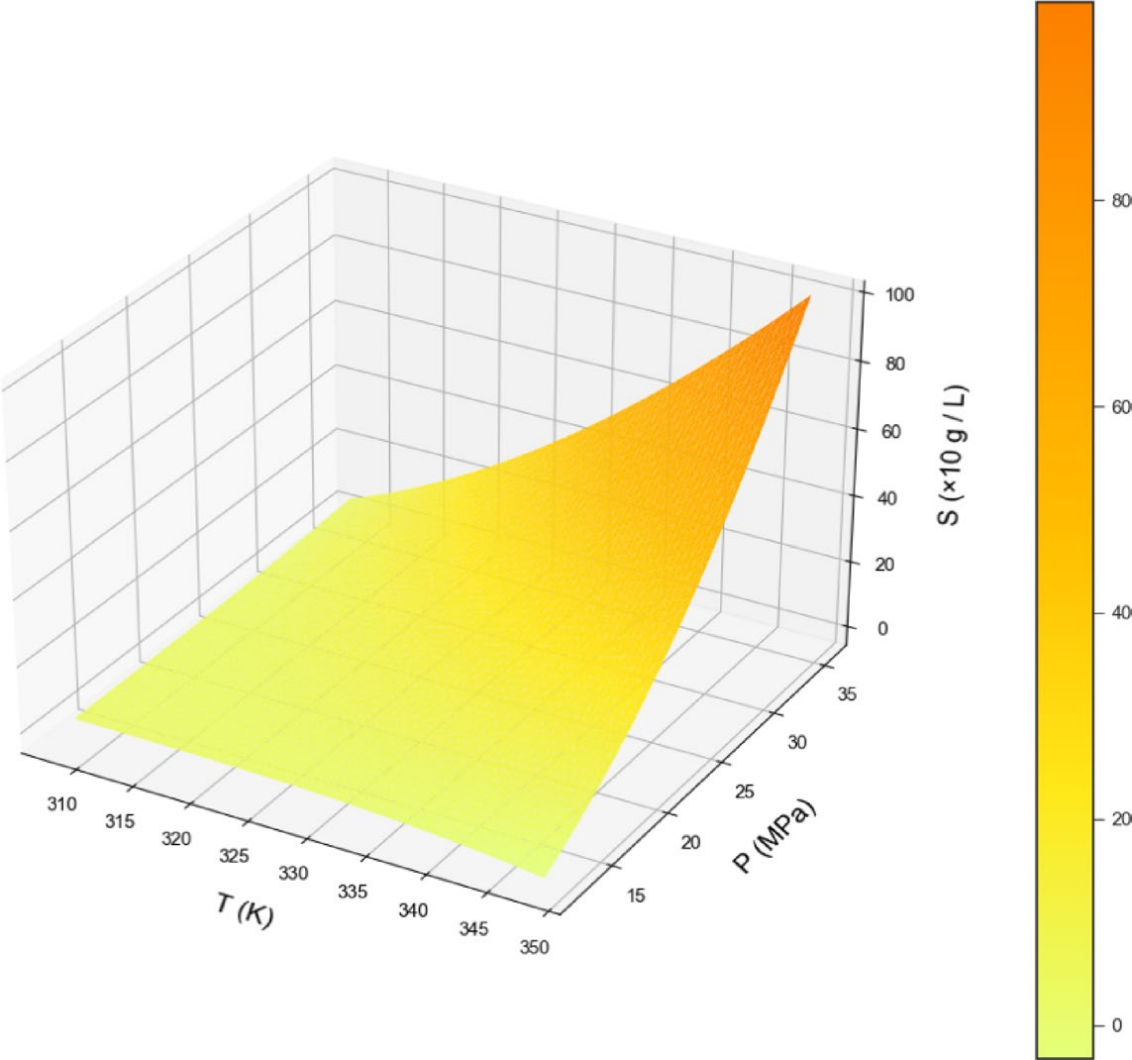
The 3D final decision surface (Nu-SVR MODEL).

By examining Table 2, the GPR estimator is the most accurate model of our work. Figures 2, 3 and 4 also show a visual comparison of the experimental values and the values obtained from the approaches. The comparison of these three figures demonstrate that the GPR model is the most accurate one and after that the US-SVR is ranked second. Also, 3D diagrams of all three final estimators are displayed in Figs. 5 and 6, and 7.

**Fig. 8:**
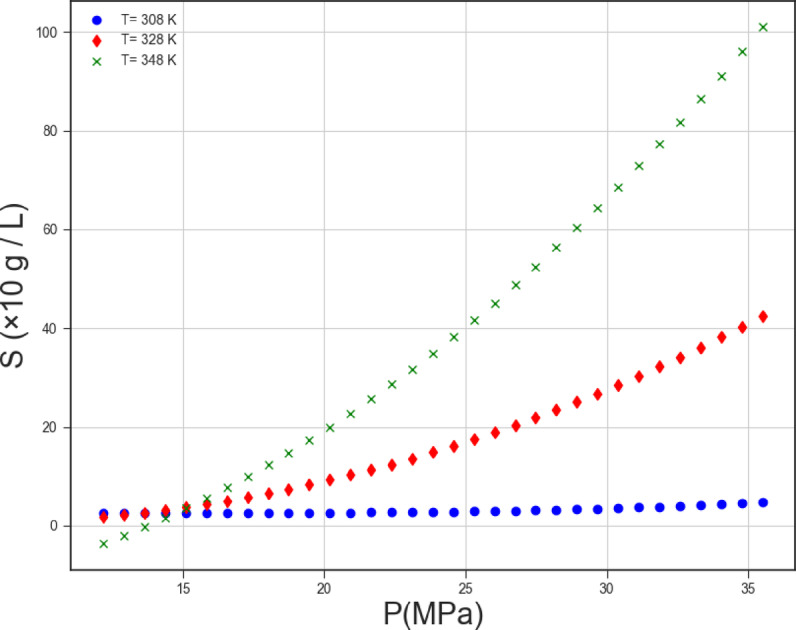
Trends of parameter P.

**Fig. 9:**
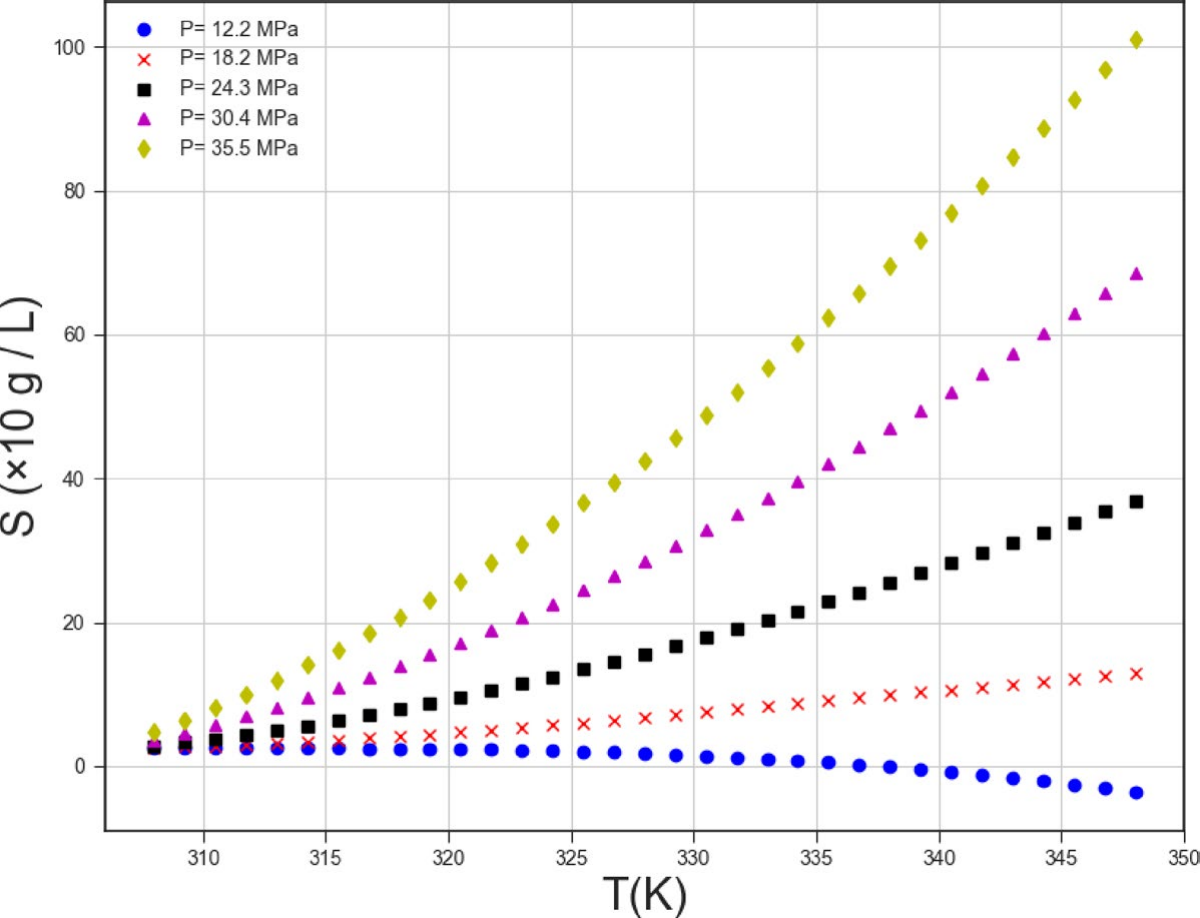
Trends of parameter T.

Based on what was said in the previous paragraph, we considered the Gaussian Process Regression model as the main model amongst others and obtained the two-dimensional trends of the parameters depicted in Figs. 8 and 9 with the help of this model. The influence of two functional parameters (pressure and temperature) on the solubility of Exemestane steroidal aromatase inhibitor anti-cancer drug is depicted in Figs. 8 and 9, respectively. Increase in the pressure is in favor of Exemestane solubility. Indeed, increase in the operating pressure dramatically improves the solvent’s density and declines intermolecular spaces between CO_2_ molecules, which positively encourages the Exemestane solubility. The influence of temperature on the solubility of Exemestane steroidal aromatase inhibitor anti-cancer drug is more complex due to the paradoxical effect of this parameter on the solute’s sublimation pressure, solvent density, and intermolecular interactions in CO_2_-SCF system. The analysis of the figures confirms that when the system pressure exceeds the cross-over point, variations in temperature significantly influence the solubility of Exemestane. This behavior arises because the positive contribution of increased sublimation pressure outweighs the adverse effect associated with the reduction in solvent density. Then, at this condition the solubility improves considerably. When the operating pressure adjusts below the cross-over value, deteriorative contribution of density reduction overcome the favorable role of increasing the sublimation pressure and thus, the solubility reduces considerably^47^.

To evaluate the generalization capability of the developed GPR model, the same modeling pipeline was applied to ten additional drug solubility datasets in supercritical CO_2_. As summarized in Table 3, the R² values ranged from 0.966 to 0.991, confirming the robustness and adaptability of the proposed model across diverse drug molecules.

**Table 3:** R^2^ performance of the GPR model for additional drug datasets.

Drug name	*R*^2^ score
Aprepitant	0.982
Docetaxel	0.977
Crizotinib	0.969
Oxycodone hydrochloride	0.985
Lansoprazole	0.975
Palbociclib	0.988
Repaglinide	0.981
Finasteride	0.979
Busulfan	0.966
Paclitaxel	0.991

## Conclusion

Identifying diverse state-of-the-art and breakthrough strategies to improve the bioavailability and solubility of orally administered anticancer agents remains a paramount concern among medical researchers. In this research study, the solubility of Exemestane steroidal aromatase inhibitor versus operating temperatures and pressures is modeled and optimized using machine learning approach. Adjusting the hyper-parameters of three separate models—NU-SVR, GPR and LASSO is done with an approach called the cuckoo search algorithm (CS), which is employed to tackle the problem of the model selection process. R-square scores of 0.996, 0.793, and 0.983 were obtained for the GPR, Nu-SVR, and LASSO models, respectively, based on the evaluations that were carried out as part of this study. The GPR model, the Nu-SVR model, and the LASSO model each exhibit MAE errors with respective values of 0.904, 5.310, and 1.921 with regard to error rate. In light of these findings and the results of the other evaluations, the Gaussian process model emerges as the model within the scope of this research as having the highest degree of precision.

## Data Availability

The data supporting this study are available when reasonably requested from the corresponding author.

## References

[1] Wairkar, S., Gaud, R. & Raghavan, A. Multi-particulate systems: cutting-edge technology for controlled drug delivery. *Recent Pat. Drug Deliv. Formul.* **10**(3), 184–191 (2016). 27809755 10.2174/1872211310666161103120006

[2] Hillery, A. & Park, K. *Drug Delivery: Fundamentals and Applications* (CRC, 2016).

[3] Garbayo, E. et al. Nanomedicine and drug delivery systems in cancer and regenerative medicine. *Wiley Interdisciplinary Reviews: Nanomed. Nanobiotechnol.* **12**(5), e1637 (2020). 10.1002/wnan.1637 32351045

[4] Baig, M. R., Shahiwala, A. & Khan, S. Sensible use of technologies to increase solubility and bioavailability in formulation development. *Advancements Bioequivalence Bioavailab.* **1**(1), 1–4 (2018).

[5] Taleghani, A. S. et al. Mesoporous silica nanoparticles as a versatile nanocarrier for cancer treatment: A review. *J. Mol. Liq.* **328**, 115417 (2021).

[6] Yasir, M. et al. Biopharmaceutical classification system: an account. *Int. J. PharmTech Res.* **2**(3), 1681–1690 (2010).

[7] Yasuji, T., Takeuchi, H. & Kawashima, Y. Particle design of poorly water-soluble drug substances using supercritical fluid technologies. *Adv. Drug Deliv. Rev.* **60**(3), 388–398 (2008). 18068261 10.1016/j.addr.2007.03.025

[8] Notej, B. et al. Increasing solubility of phenytoin and raloxifene drugs: application of supercritical CO_2_ technology. *J. Mol. Liq.* 121246 (2023).

[9] Alshahrani, S. M. et al. Measurement of metoprolol solubility in supercritical carbon dioxide; experimental and modeling study. *Case Stud. Therm. Eng.* 102764 (2023).

[10] Tran, P. & Park, J. S. Application of supercritical fluid technology for solid dispersion to enhance solubility and bioavailability of poorly water-soluble drugs. *Int. J. Pharm.* **610**, 121247 (2021). 34740762 10.1016/j.ijpharm.2021.121247

[11] Cao, Y. et al. Recent advancements in molecular separation of gases using microporous membrane systems: A comprehensive review on the applied liquid absorbents. *J. Mol. Liq.* **337**, 116439 (2021).

[12] Cheng, Z. et al. Post-combustion CO2 capture and separation in flue gas based on hydrate technology: A review. *Renew. Sustain. Energy Rev.* **154**, 111806 (2022).

[13] Alzhrani, R. M., Almalki, A. H. & Alshehri, S. Novel numerical simulation of drug solubility in supercritical CO2 using machine learning technique: Lenalidomide case study. *Arab. J. Chem.* **15**(11), 104180 (2022).

[14] Azim, M. M. et al. Modeling the solubility of non-steroidal anti-inflammatory drugs (ibuprofen and ketoprofen) in supercritical CO2 using PC-SAFT. *J. Supercrit. Fluids*. **186**, 105626 (2022).

[15] Goss, P. E. et al. Exemestane for breast-cancer prevention in postmenopausal women. *N. Engl. J. Med.* **364**(25), 2381–2391 (2011). 21639806 10.1056/NEJMoa1103507

[16] Scott, L. J. & Wiseman, L. R. Exemestane. *Drugs*. **58**, 675–680 (1999). 10551437 10.2165/00003495-199958040-00007

[17] Chaturvedi, S. & Garg, A. A comprehensive review on novel delivery approaches for exemestane. *J. Drug Deliv. Sci. Technol.* 103655 (2022).

[18] Shang, Y. et al. Artificial neural network hyperparameters optimization for predicting the thermal conductivity of MXene/graphene nanofluids. *J. Taiwan Inst. Chem. Eng.* **164**, 105673 (2024).

[19] Graish, M. S. et al. Prediction of the viscosity of iron-CuO/water-ethylene glycol non-Newtonian hybrid nanofluids using different machine learning algorithms. *Case Stud. Chem. Environ. Eng.* **11**, 101180 (2025).

[20] Ismail, M. A. et al. Machine learning-based optimization and dynamic performance analysis of a hybrid geothermal-solar multi-output system for electricity, cooling, desalinated water, and hydrogen production: A case study. *App. Therm. Eng.* **267**, 125834 (2025).

[21] Togun, H. et al. Advancing organic photovoltaic cells for a sustainable future: the role of artificial intelligence (AI) and deep learning (DL) in enhancing performance and innovation. *Sol. Energy*. **291**, 113378 (2025).

[22] Hai, T. et al. Optimizing ternary hybrid nanofluids using neural networks, gene expression programming, and multi-objective particle swarm optimization: a computational intelligence strategy. *Sci. Rep.* **15**(1), p1986 (2025). 10.1038/s41598-025-85236-3 PMC11736119 39814861

[23] Bhatt, D. et al. An enhanced mems error modeling approach based on nu-support vector regression. *Sensors* **12**(7), 9448–9466 (2012). 23012552 10.3390/s120709448 PMC3444110

[24] Martin, M. On-line support vector machine regression. In *European Conference on Machine Learning*. (Springer, 2002).

[25] Alqarni, M. et al. Solubility optimization of loxoprofen as a nonsteroidal anti-inflammatory drug: statistical modeling and optimization. *Molecules* **27**(14), 4357 (2022). 35889230 10.3390/molecules27144357 PMC9321224

[26] Gershman, S. J. & Blei, D. M. A tutorial on Bayesian nonparametric models. *J. Math. Psychol.* **56**(1), 1–12 (2012).

[27] Williams, C. K. Prediction with Gaussian processes: from linear regression to linear prediction and beyond. In *Learning in Graphical Models* 599–621 (Springer, 1998).

[28] Schulz, E., Speekenbrink, M. & Krause, A. A tutorial on Gaussian process regression: Modelling, exploring, and exploiting functions. *J. Math. Psychol.* **85**, 1–16 (2018).

[29] Hoang, N. D. et al. Estimating compressive strength of high performance concrete with Gaussian process regression model. *Adv. Civil Eng.* **2016** (2016).

[30] Kim, C. et al. Case influence diagnostics in the Lasso regression. *J. Korean Stat. Soc.* **44**(2), 271–279 (2015).

[31] Hojjati, M. et al. Supercritical CO_*2*_ and highly selective aromatase inhibitors: Experimental solubility and empirical data correlation. *J. Supercrit. Fluids*. **50**(3), 203–209 (2009).

[32] Chakraborty, S. & Mali, K. Biomedical image segmentation using fuzzy multilevel soft thresholding system coupled modified cuckoo search. *Biomed. Signal Process. Control*. **72**, 103324 (2022).

[33] Yang, X. S. & Deb, S. Cuckoo Search Via Lévy flights. In *2009 World Congress on Nature & Biologically Inspired Computing (NaBIC)* (IEEE, 2009).

[34] Liu, Y. et al. Machine learning based modeling for estimation of drug solubility in supercritical fluid by adjusting important parameters. *Chemometr. Intell. Lab. Syst.* **254**, 105241 (2024).

[35] Wang, Y. et al. Prognostic staging of esophageal cancer based on prognosis index and cuckoo search algorithm-support vector machine. *Biomed. Signal Process. Control*. **79**, 104207 (2023).

[36] Obaidullah, A. J. & Almehizia, A. A. Machine learning-based prediction and mathematical optimization of capecitabine solubility through the supercritical CO2 system. *J. Mol. Liq.* **391**, 123229 (2023).

[37] Grbić, R., Kurtagić, D. & Slišković, D. Stream water temperature prediction based on Gaussian process regression. *Expert Syst. Appl.* **40**(18), 7407–7414 (2013).

[38] Rasmussen, C. E. Gaussian processes in machine learning. In *Summer School on Machine Learning* (Springer, 2003).

[39] Jin, H. et al. Computational simulation using machine learning models in prediction of CO_2_ absorption in environmental applications. *J. Mol. Liq.* **358**, 119159 (2022).

[40] Lu, Y. et al. Molecular separation and computational simulation of contaminant removal from wastewater using zirconium UiO-66-(CO2H) 2 metal–organic framework. *J. Mol. Liq.* **365**, 120178 (2022).

[41] Rasmussen, C. E. & Nickisch, H. Gaussian processes for machine learning (GPML) toolbox. *J. Mach. Learn. Res.* **11**, 3011–3015 (2010).

[42] Drucker, H. et al. Support vector regression machines. *Adv. Neural. Inf. Process. Syst.* **9** (1996).

[43] Müller, K. R. et al. Predicting time series with support vector machines. In *International Conference on Artificial Neural Networks*. (Springer, 1997).

[44] Cortes, C. & Vapnik, V. Support-vector networks. *Mach. Learn.* **20**(3), 273–297 (1995).

[45] Catoni, O. Challenging the empirical mean and empirical variance: a deviation study. In *Annales de l’IHP Probabilités et statistiques*. (2012).

[46] Naidu, G., Zuva, T. & Sibanda, E. M. A review of evaluation metrics in machine learning algorithms. In *Computer Science On-line Conference*. (Springer, 2023).

[47] Alamri, A. & Alafnan, A. Artificial intelligence optimization of Alendronate solubility in CO_2_ supercritical system: Computational modeling and predictive simulation. *Ain Shams Eng. J.* **15**(9), 102905 (2024).

